# Experimental Shape Sensing and Load Identification on a Stiffened Panel: A Comparative Study

**DOI:** 10.3390/s22031064

**Published:** 2022-01-29

**Authors:** Marco Esposito, Massimiliano Mattone, Marco Gherlone

**Affiliations:** Department of Mechanical and Aerospace Engineering, Politecnico di Torino, 10129 Torino, Italy; marco.esposito@polito.it (M.E.); massimiliano.mattone@polito.it (M.M.)

**Keywords:** experimental testing, shape sensing, load identification, stiffened panel, aerospace structure, structural health monitoring, SHM, displacements reconstruction, iFEM, strain

## Abstract

The monitoring of loads and displacements during service life is proving to be crucial for developing a modern Structural Health Monitoring framework. The continuous monitoring of these physical quantities can provide fundamental information on the actual health status of the structure and can accurately guide pro-active condition-based maintenance operations, thus reducing the maintenance costs and extending the service life of the monitored structures. Pushed by these needs and by the simultaneous development in the field of strain sensing technologies, several displacement reconstruction and load identification methods have been developed that are based on discrete strain measurements. Among the different formulations, the inverse Finite Element Method (iFEM), the Modal Method (MM) and the 2-step method, the latter being the only one able to also compute the loads together with the displacements, have emerged as the most accurate and reliable ones. In this paper, the formulation of the three methods is summarized in order to set the numerical framework for a comparative study. The three methods are tested on the reconstruction of the external load and of the displacement field of a stiffened aluminium plate starting from experimentally measured strains. A fibre optic sensing system has been used to measure surface strains and an optimization procedure has been performed to provide the best fibre pattern, based on five lines running along the stiffeners’ direction and with a back-to-back measuring scheme. Additional sensors are used to measure the applied force and the plate’s deflection in some locations. The comparison of the results obtained by each method proves the extreme accuracy and reliability of the iFEM in the reconstruction of the deformed shape of the panel. On the other hand, the Modal Method leads to a good reconstruction of the displacements, but also exhibits a sensitivity to the choice of the modes considered for the specific application. Finally, the 2-step approach is able to correctly identify the loads and to reconstruct the displacements with an accuracy that depends on the modeling of the experimental setup.

## 1. Introduction

The Structural Health Monitoring (SHM) is rapidly growing as one of the most relevant fields of research for the improvement of the performance of the structures during their service life. In particular, the SHM framework is rapidly changing its traditional formulation, based on the pre-emptive maintenance, to a new principle based on pro-active condition-based maintenance. This change of paradigm strongly relies on the possibility to continuously monitor some physical quantities that can give fundamental information on the status of the structure. Among them, loads and displacements can lead to important evaluations on the status of the structure [1,2,3,4,5] and can accurately guide the structural maintenance operations [6], thus consistently reducing the costs by avoiding unnecessary scheduled interventions and increasing the safety by determining critical conditions that require immediate actions. Moreover, the on-line monitoring of these quantities is crucial for the control of the morphing structures. In fact, the knowledge of the loads and displacements can guide their morphing mechanism to obtain load alleviation and to improve the aerodynamic efficiency of the control surfaces [7,8].

The direct monitoring of these quantities through dedicated sensors is complex and sometime, especially for aerospace structures, even impossible due to the difficulties connected with their installation. On the other hand, the strains are quantities that are easily measurable on a structure and, recently, with the development of fibre optics distributed strain sensing systems [9,10], the availability of a considerable quantity of stain information with the use of less invasive sensors has even increased. For these reasons, indirect methods that can compute the loads and displacements from discrete stain measurements have seen a rapid development.

The displacement reconstruction from discrete strain measurements is often defined as shape sensing. Several shape sensing methods, based on different principles have been designed, including the methods based on the numerical integration of the strain measurements and the Bernoulli-Euler beam theory [11,12,13], the methods based on the use of the modal shapes and the modal strain shapes [14,15,16], the methods based on an inverse formulation of the Finite Element Method (FEM) and the methods based on the use of Artificial Neural Networks (ANNs) [17,18]. A detailed review of the existing shape sensing methods can be found in [19]. Among the given formulations, the Modal Method (MM) and the inverse Finite Element Method (iFEM) have emerged as the most accurate and also robust with respect to the uncertainties of the inputs typical of experimental scenarios [20,21].

The MM is based on expressing displacements and strains in terms of modal shapes and modal coordinates [14,22]. The modal coordinates are then computed by fitting the strain field to discrete measured strains. In [14], experimentally measured modal characteristics were used to predict the static deformation of an aluminium cantilevered plate. The influence of the number and position of the strain sensors was also considered in the study. In [15], the shape sensing of a cantilevered plate was performed using the modal characteristics computed numerically through a FE model of the structure. The investigation also included a criterion for the selection of the modes for the application of the method. The criterion is based on the evaluation of the strain energy of each mode. Recently, the MM has also been applied on the static and dynamic analyses of a wing and a different criterion for the selection of the modes, based on orthogonality considerations, has been formulated [16].

The iFEM is based on the discretisation of the structural domain with Finite Elements and on the formulation of an error functional [23]. This functional expresses the error between the strain field measured at some discrete locations and the analytical one expressed in terms of the nodal degrees of freedom through the FE discretisation. The minimisation of this functional allows the computation of the displacements that best fit the measured strain field. Several formulations of the method have been proposed, based on the definition of different inverse elements. Beam elements have been formulated for the monitoring of truss and beam structures [24,25,26,27]. Three-nodes inverse shell elements have been widely used for the analysis of thin plates [28,29] and thin walled structures [30,31]. A triangular element based on the refined zig-zag theory has been recently presented in [32] for the shape sensing of multilayered composite and sandwich structures. Quadrilateral inverse shell elements have been developed and applied using standard [33,34,35] and isogeometric [36] formulations. Recently, the iFEM has been experimentally applied to the full displacement field reconstruction of an aeronautical stiffened panel in [37].

The research on the load identification methods from discrete strain measurements has been mainly developed within the analysis of aerospace structures. Shkarayev et al. [38] developed a method based on the parametric approximation of the aerodynamic loading. The load is expressed as a combination of known distributions and the strains induced by these distributions are compared with the measured ones in order to determine the weight of each known distribution to the combination approximating the applied load. Cameron et al., extended the method by adopting single Fourier cosine terms [39] and double Fourier series [40] to parametrize different two-variables pressure distributions over a square plate. A method based on the coefficients of influence and on the discretisation of an applied pressure field with FE has been introduced in [6]. The coefficients of influence are obtained by computing the strain field induced by each discrete load. The unknown discrete values of the load are computed by fitting the strain field caused by each discrete load to the measured strains. In [41], the effect of the regularization of the solution for cases affected by measurement errors has been studied for this approach. This method inspired the formulation of a new shape sensing method based on a 2-step approach [42,43]. According to this proposed formulation, the identification of the load represents the first step of the 2-step procedure. During the 2nd step, the computed loads are used to calculate the displacements by means of a detailed FE model of the structure. The method is able to simultaneously monitor the loads and displacements of a structure using the same measured strains. In the works that introduced the procedure, the numerical verification of the method on two aeronautical applications has been performed, but no experimental assessment of the method has been presented so far. Therefore, an experimental validation of the method is still required for the further evaluation of this technology.

Few comparative studies relative to the introduced methods are available in the open literature. In [19] the MM and the iFEM are compared on the experimental monitoring of a cantilevered wing-shaped plate, highlighting the superior accuracy of the iFEM. The two methods are also compared in [20,21], this time on a numerical composite wing box. These studies reveal a good accuracy of the MM when few strain sensors are available but a superior accuracy of the iFEM when more sensors are used. In [43], the shape sensing capabilities of the 2-step method are compared with the ones of the iFEM on the numerical analysis of an aluminium wing box. For this numerical application the 2-step method results more accurate than the iFEM for different configurations of strain sensors. Nevertheless, the accurate evaluation of these numerical results still needs the support of an experimental validation. The listed comparative studies only compare a combination of two out of the three introduced methods at a time.

In this paper, for the first time, the three shape sensing methods, i.e., the MM, the iFEM and the 2-step approach, are compared on the experimental monitoring of a standard aerospace component, an aluminium stiffened panel. This study is fundamental for the evaluation of the performances and drawbacks of each method in an experimental scenario. Moreover, it is also important for the assessment of the different characteristics that make each method suited for a specific application, thus providing information on the best monitoring method according to its peculiarities. This work also presents the first experimental application of the 2-step approach for the simultaneous reconstruction of the applied loads and of the displacement field of the structure. This experimental study proves the superior accuracy of the iFEM with respect to the other two methods, that are nevertheless capable of a good reconstruction of the deformed shape of the panel. Moreover, this work highlights the sensitivity of the MM to the selection of the modes retained during the analysis and of the 2-step approach to the capability of the refined model to capture the real behaviour of the monitored structure.

The paper is structured as follows. In Section 2, the three methods are introduced. In Section 3, the experimental setup, the models and the preliminary operations assessed before performing the test are described. The application of the three methods to the experimental test and the comparison of the results is discussed in Section 4. Finally, the concluding remarks are presented in Section 5.

## 2. Methods

In this section the shape sensing methods, as they will be used throughout this publication, are briefly described. The three described methods are all based on the reconstruction of the displacement field from discrete strain measurements and they differ from one another because of the principles that they are based on.

### 2.1. The Modal Method

The Modal Method (MM) uses the modal shapes as basis functions to describe the displacement field [14]. To correlate the displacement field, expressed in terms of the modal shapes, to the measured strains, the strain-displacement equation is adopted. Considering a FE discretization of the structural domain, the procedure is based on the expression of the displacement degrees of freedom and of the strain components in terms of the modal matrices and of the modal coordinates (q):

(1) $$w=\Phi_{d}\;q$$

(2) $$\varepsilon =\Phi_{s}\;q$$

where $w_{D\times 1}$ is the nodal degrees-of-freedom (DOFs) vector and $\varepsilon_{S\times 1}$ is the strain vector. The modal matrix ${\left[\Phi_{d}\right]}_{D\times M}$ is constituted by M columns (the *i*-th column being the *i*-th modal eigenvector of the degrees-of-freedom). The modal matrix ${\left[\Phi_{s}\right]}_{S\times M}$ is also constituted by M columns (the *i*-th column being the *i*-th set of strains corresponding to the *i*-th mode shape of the FE model of the structure). By inverting Equation (2), in case of a squared $\Phi_{s}$ matrix, the expression of the modal coordinates in terms of ε is obtained:

(3) $$q=\Phi_{s}^{-1}\;\varepsilon$$

Then, substituting Equation (3) into Equation (1) leads to the expression of the nodal DOFs vector in terms of the modal matrices and the strain vector:

(4) $$w=\Phi_{d}\;\Phi_{s}^{-1}\;\varepsilon$$

The application of Equation (4) for the computation of a set of $D_{c}$ displacement components ($w_{D_{c}\times 1}^{c}$), in real world scenarios, implies that the strain vector ε is substituted with the vector of the measured strain components (${\varepsilon^{m}}_{S_{m}\times 1}$), coming form a set of $S_{m}$ sensors. Moreover, a limited number of retained $M_{r}$ modes is considered. The modal matrices are modified accordingly, to consider the rows only relative to the considered quantities and the columns relative to the retained modes, thus resulting in ${\left[\Phi_{s}^{m}\right]}_{S_{m}\times M_{r}}$ and ${\left[\Phi_{d}^{c}\right]}_{D_{c}\times M_{r}}$. Since the number of retained modes and the number of measured strain components are usually different, this also leads to having not-squared ${\left[\Phi_{s}^{m}\right]}_{S_{m}\times M_{r}}$ matrix. Therefore, the Moore-Penrose pseudo inverse formulation [44], ${\left(\Phi_{s}^{m}\right)}^{+}$, can be adopted to obtain the generalisation of Equation (4) for non-squared matrices, when considering a set of displacement components, a set of actually measured strains and a reduced number of retained modes:

(5) $$w^{c}=\Phi_{d}^{c}\;{\left(\Phi_{s}^{m}\right)}^{+}\;\varepsilon^{m}$$

The selection of the modes that contribute the most to the description of the deformed shape of the investigated structure can be evaluated with the selection criterion, inspired by energy contribution evaluations, described in [15,20].

The MM requires the computation of the mode shapes of the structure. Therefore it needs the knowledge of the material characteristics, that influence them.

### 2.2. The Inverse Finite Element Method

The iFEM is a shape sensing method based on a finite element discretisation of the structural domain [23]. In particular, for thin-walled structures, the formulation based on the inverse four-node shell finite elements is considered. The First Order Shear Deformation Theory (FSDT) for thin plates is adopted to define the kinematic behaviour of the structure [45]. According to the theory, the strain field can be expressed in terms of the plate’s reference surface in-plane displacements, u and v, the transverse displacement, w, and the rotations around the mid-plane axes x and y, $\theta_{x}$ and $\theta_{y}$, as:

(6a) $$\begin{array}{cc} \; & \left\{\begin{array}{c} \varepsilon_{\mathrm{xx}}\;\\ \varepsilon_{\mathrm{yy}}\;\\ \gamma_{\mathrm{xy}}\; \end{array}\right\}=\left\{\begin{array}{c} u_{,x}\;\\ v_{,x}\;\\ v_{,x}+u_{,y}\; \end{array}\right\}+z\left\{\begin{array}{c} \theta_{y,x}\;\\ -\theta_{x,y}\;\\ (\theta_{y,y}-\theta_{x,x})\; \end{array}\right\}=\left\{\begin{array}{c} \varepsilon_{1}\;\\ \varepsilon_{2}\;\\ \varepsilon_{3}\; \end{array}\right\}+z\left\{\begin{array}{c} \varepsilon_{4}\;\\ \varepsilon_{5}\;\\ \varepsilon_{6}\; \end{array}\right\} \end{array}$$

(6b) $$\begin{array}{cc} \; & \left\{\begin{array}{c} \gamma_{\mathrm{xz}}\;\\ \gamma_{\mathrm{yz}}\; \end{array}\right\}=\left\{\begin{array}{c} w_{,x}+\theta_{y}\;\\ w_{,y}-\theta_{x}\; \end{array}\right\}=\left\{\begin{array}{c} \varepsilon_{7}\;\\ \varepsilon_{8}\; \end{array}\right\} \end{array}$$

where *z* is the thickness coordinate. Therefore, the strain field of the FSDT can be fully described by eight strain measures: $\varepsilon_{k}\;\left(k=1,2,3\right)$ represent the membrane strain measures, $\varepsilon_{k}\;\left(k=4,5,6\right)$ are the bending curvatures and $\varepsilon_{k}\;\left(k=7,8\right)$ are the transverse shear strains of the plate.

The introduction of the FE discretisation leads to the expression of the kinematic variables, within each element, in terms of the shape functions, N, and of the element’s nodal degrees of freedom (DOFs), $u^{e}$:

(7) $${\left[u,\;v,\;w,\;\theta_{x},\;\theta_{y}\right]}^{T}=Nu^{e}\;\;\;\;\;\;$$

In this paper, the inverse four-node iQS4 element’s formulation is adopted. The expression of the shape functions for iQS4 can be found in [35]. By substituting Equation (7) into Equations (6), it is possible to express the strain measures in terms of the nodal DOFs:

(8) $$\varepsilon_{k}\left(u^{e}\right)=B_{k}u^{e}\;\;\;\;\left(k=1,\;2,\;...,8\right)$$

where $B_{k}$ is the matrix containing the spatial derivatives of the shape functions corresponding to the *k*-th strain measure.

The working principle of the inverse Finite Element Method is to find the nodal DOF values that minimize the error between the strain measures, as defined in Equation (8), and the ones experimentally measured in a finite number of discrete locations. The error within each element is expressed through the least-square functional:

(9) $$\Psi^{e}\left(u^{e}\right)=\sum_{k=1}^{8}\lambda_{k}^{e}w_{k}^{e}{\;\int \int \;}_{A^{e}}{\left(\varepsilon_{k}\left(u^{e}\right)-\varepsilon_{k}^{m}\right)}^{2}\mathrm{dxdy}$$

where $\varepsilon_{k}^{m}$ is the value of the *k*-th experimental strain measure within the element. The first six strain measures can be easily obtained from strain sensors located on the bottom and top surface of the plate [29] whereas transverse shear strains are not experimentally measurable. The terms $w_{k}^{e}$ are the dimensional coefficients required to guarantee the physical units consistency of Equation (9). They are set as follows: $w_{k}^{e}=1$ for k=1,2,3,7,8 and $w_{k}^{e}={\left(2h\right)}^{2}$ for k=4,5,6, where h is the half-thickness of the element. The terms $\lambda_{k}^{e}$ are the penalization factors that allow to take into account for the absence of a measured strain. In fact, they are set to **1** when the corresponding strain is measured or to a small value ($10^{-4},10^{-5},10^{-6}$) when it is not measured. In the last case, the corresponding $\varepsilon_{k}^{m}$ is set to 0 (as a consequence, the terms $\lambda_{7,8}^{e}$ are always set to a small value and the terms $\varepsilon_{7,8}^{\varepsilon }$ are always set to 0). The integral over the area of the element, $A^{e}$, in Equation (9), is numerically computed using Gaussian quadrature. Therefore, it is transformed into a summation over the n×n quadrature points:

(10) $${\;\int \int \;}_{A^{e}}{\left(\varepsilon_{k}\left(u^{e}\right)-\varepsilon_{k}^{\varepsilon }\right)}^{2}\mathrm{dxdy}=\sum_{g=1}^{n\times n}J\left(g\right)\omega_{g}{\left(\varepsilon_{k(g)}\left(u^{e}\right)-\varepsilon_{k(g)}^{m}\right)}^{2}\;\;\;\left(k=1,\;2,\;...,8\right)$$

where $\omega_{g}$ are the quadrature weights, and J(g) is the determinant of the Jacobian of the transformation from the physical coordinates to the natural ones of the element computed in the *g*-th quadrature point. The subscript g denotes the computation of the quantity in the *g*-th quadrature point.

The minimisation of the error functional, Equation (9), with respect to the nodal DOFs, $u^{e}$, leads to the solution of a system of linear equations:

(11a) $$\begin{array}{cc} \; & \frac{\partial \Psi_{e}\left(u^{e}\right)}{\partial u^{e}}=l^{e}u^{e}-f^{e}=0 \end{array}$$

(11b) $$\begin{array}{cc} \; & u^{e}={l^{e}}^{-1}f^{e} \end{array}$$

The assembly procedure, typical of the standard FEM, is then adopted to extend the procedure to all the elements of the structure. As a consequence, the assembly of the $l^{e}$ matrices generates the global L matrix and the assembly of the $f^{e}$ vectors generates the global F vector. The vector of the global DOFs, U, can then be computed as:

(12) $$U=L^{-1}F$$

The complete expressions of the $l^{e}$ and $f^{e}$ matrices for the iQS4 element are:

(13a) $$\begin{array}{cc} \; & l^{e}=\sum_{k=1}^{8}\sum_{g=1}^{n\times n}\left[J\left(g\right)\lambda_{k}^{e}w_{k}^{e}\omega_{g}\chi_{g}B_{k(g)}^{T}B_{k(g)}\right]\;\;\;\;\;\;\;\;\left(\begin{array}{c} \chi_{g=\mathrm{centroid}}=1\\ \chi_{g\neq \mathrm{centroid}}=10^{-4} \end{array}\right) \end{array}$$

(13b) $$\begin{array}{cc} \; & f^{e}=\sum_{k=1}^{6}\sum_{g=1}^{n\times n}\left[J\left(g\right)\lambda_{k}^{e}w_{k}^{e}\omega_{g}\chi_{g}B_{k(g)}^{T}\varepsilon_{k(\mathrm{centroid})}^{m}\right]\;\;\left(\begin{array}{c} \chi_{g=\mathrm{centroid}}=1\\ \chi_{g\neq \mathrm{centroid}}=10^{-4} \end{array}\right) \end{array}$$

where a further penalization scheme is introduced (similar to the one adopted to take into account missing strain measures [20]). Considering the difficulty to set n×n measurement points inside an inverse element, the assumption is made that at least the centroidal experimental measure, $\varepsilon_{k(\mathrm{centroid})}^{m}$, is available (the penalisation coefficient $\chi_{g}$ is set to 1 for that Gauss point) and that the strain measure is uniform over the element area (but with $\chi_{g}$ set to $10^{-4}$ for the other Gauss points). This penalisation scheme is introduced for elements where a strain measure is present. As a consequence, for k=7,8 and for elements without sensors at all, only the penalization strategy introduced by $\lambda_{k}^{e}$ is adopted.

The iFEM is only based on the strain-displacement relationship and, therefore, does not require any knowledge of the material characteristics of the monitored structure.

### 2.3. The 2-Step Method

This method is based on the identification of the external loads from discrete strain measurements. The identified loads are then applied to a FE model of the structure to obtain the displacement field, thus simultaneously computing the loads and the deformed shape of the structure.

The identification of the loads is based on the discretisation of the load system into discrete components. In [43] the formulation for concentrated loads and distributed ones has been derived. However, since for this application only concentrated loads are considered, only this case is presented. In the linear elastic regime, the strain field induced by a system of discrete loads can be expressed as a superposition of the strain fields induced by each *i*-th discrete load, $F_{i}$. As a consequence, the *j*-th strain component, $\varepsilon_{j}$, can be expressed by the superposition of the strains induced by the $m_{l}$ loads:

(14) $$\varepsilon_{j}=\sum_{i=1}^{m_{l}}\varepsilon_{\mathrm{ji}}=\sum_{i=1}^{m_{l}}s_{\mathrm{ji}}\;F_{i}$$

where $s_{\mathrm{ji}}$ is an unknown influence coefficient that relates the *j*-th strain component to the *i*-th discrete value of the external load. Considering a vector of a finite number ($S_{m}$) of measured strain components, $\varepsilon_{S_{m}\times 1}^{m}$, and expressing Equation (14) in matrix form leads to:

(15) $$\varepsilon^{m}=S\;F$$

where $F_{m_{l}\times 1}$ is the vector of the discrete loads. The ${\left[S\right]}_{S_{m}\times m_{l}}$ is the matrix of the influence coefficients between the strains and the loads. If a FE model of the analysed structure is available, ${\left[S\right]}_{S_{m}\times m_{l}}$ can be easily populated through an iterative solution of the standard FE problem. The *i*-th column of the matrix is computed by imposing that $F_{i}=1$ and $F_{k}=0\;\left(k\neq i\right)$. The *i*-th load is then applied to the FE model of the structure and the desired $S_{m}$ strain components are calculated. Iterating the procedure to the $m_{l}$ loads, allows the computation of the entire matrix.

The inversion of Equation (15), by means of the generalised pseudo-inverse formulation, ${\left(S\right)}^{+}$, allows the computation of the discrete loads from discrete strain measurements:

(16) $$F={\left(S\right)}^{+}\;\varepsilon^{m}$$

The load identification represents the fist step of the 2-step procedure. The second step is related to the shape sensing. The displacements are simply reconstructed by applying the identified system of loads to a detailed FE model of the structure and by solving the standard (or direct) FEM problem, that allows the computation of the displacement when loads, material characteristics and boundary conditions are known. Previous works [42,43] have numerically proven that the second step is really accurate, even when the loads are not identified properly. In fact, the method is capable of identifying an equivalent system of loads that, although different from the actually applied one, induces the same strain and displacement fields, thus allowing the method to accurately reconstruct the deformed shape of the structure.

## 3. Experimental Setup and Preliminary Computations

### 3.1. Experimental Setup

**Figure 1:**
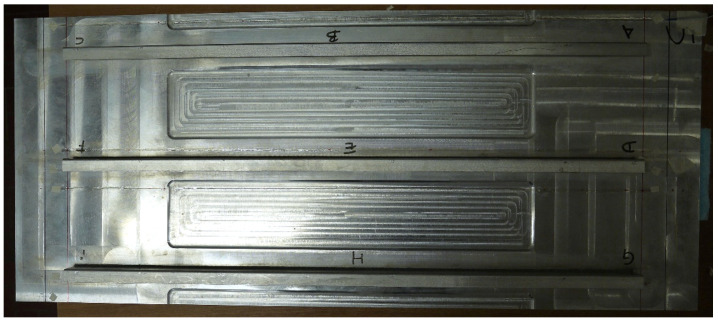
Top view of the stiffened panel.

**Figure 2:**
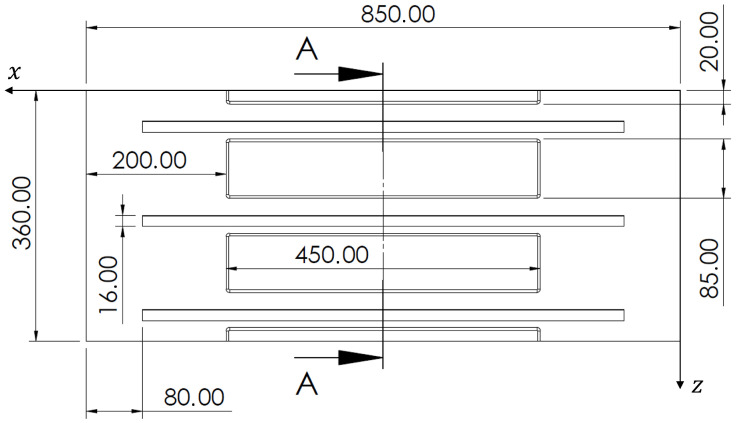
Geometry—Top view with all the dimensions in mm.

**Figure 3:**
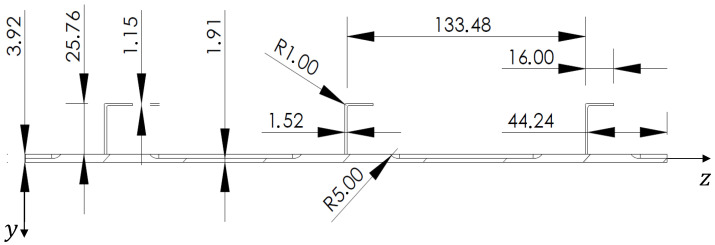
Geometry—Section A with all the dimensions in mm.

The structural component object of this investigation is typical of aerospace applications. A stiffened panel with three L-shaped stringers has been considered (Figure 1). The geometry of the panel is presented in Figure 2 and Figure 3, along with the adopted reference coordinate frame. The panel is flat, 3.92 mm thick but with a geometric complexity represented by thinner skin rectangular areas (1.91 mm) located within the individual bays defined by the stiffeners. Stringers are directly welded on the panel, thus avoiding the use of bolts and rivets, since the panel is made of an Aluminium-Lithium alloy that allows welding. The properties of the alloy are listed in Table 1.

**Figure 4:**
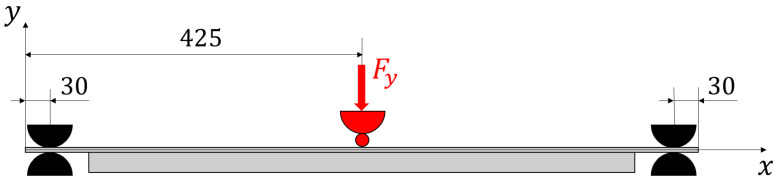
Scheme of the loading and boundary conditions with all dimension in mm.

**Figure 5:**
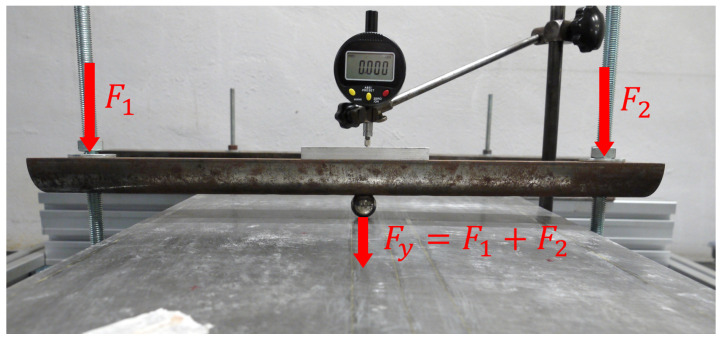
Detail of the load application system.

**Figure 6:**
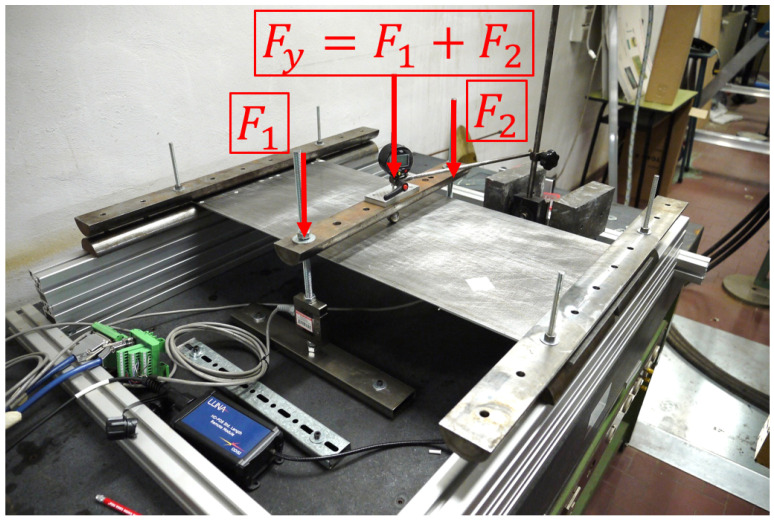
Testing configuration.

The testing configuration is based on simply supported boundary conditions for all the points located at (x=30 mm) and (x=820 mm), in the region where the panel is free from the stiffeners. The loading condition is constituted by a concentrated force ($F_{y}$) applied at the centre point (x=425 mm, z=180 mm) on the unstiffened side of the panel and oriented along the negative direction of the y axis (Figure 4). This configuration is obtained by means of half-cylindrical iron bars. The supported edges of the plate are placed between two bars tightened together with the curved side touching the panel in order to prevent transverse deflection but to allow bending rotation (Figure 4). The load is transmitted to the panel by an iron sphere. On top of the sphere, another half-cylindrical iron bar is placed that is connected, through two threaded bars and two load cells, to the test table. The panel is loaded by tightening the two nuts on the threaded bars, so that the nuts push down the half cylinder. When the bar is pushed down, the sphere transmits the load to the centre point of the panel (Figure 5). The loads generated by the nuts are measured by the two load cells. The resultant concentrated force, applied at the centre of the plate, is the sum of the two measured loads. The test configuration is presented in Figure 6.

### 3.2. Models

Two numerical models of the test configuration have been developed. The first one is the inverse Finite Element model, necessary for the application of the relative shape sensing method. The second one is a refined FE model for the computation of the modal characteristics of the structure, necessary to the MM, and for the computation of the S matrix, relative to the 2-step method. The latter model has also been adopted to generate the strains and displacements for the preliminary numerical application of the three methods and to seek the optimal sensors’ configuration.

**Figure 7:**
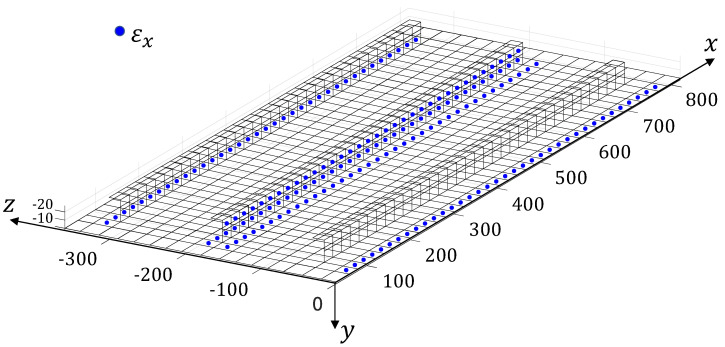
The iFEM mesh and strain sensors’ configuration.

The inverse model is constituted by 914 iQS4 elements and 978 nodes, not modelling the parts of the panel that exceed the supports (Figure 7). The refined model, used to compute the matrices for the Modal Method and the 2-step method, is obtained from the inverse one by splitting each element into four QUAD4 elements. The analysis on this model has been performed using the software MSC/NASTRAN 2017^®^.

### 3.3. Configuration of Sensors

The three methods, i.e., the iFEM, the MM and the 2-step method, are influenced by the location and quantity of the strains that are used as inputs. Therefore, the strain sensors’ configuration has been numerically optimised before conducting the experimental campaign. The optimisation process has been performed considering the sensing technology adopted experimentally, the LUNA^®^ high-definition distributed fibre optic strain sensing system. The sensor is based on Rayleigh scattering and Optical Frequency Domain Reflectometry (OFDR) [9,10] and allows the measurement of the strain component along the fibre optic direction with an impressive density. Considering a 10 m long fibre, it is possible to measure the strain for every 1.3 mm. The use of this kind of fibre allows to follow complex paths on the structure and, in the case of the stiffened panel, to measure the strain along the x direction on five sensing lines along the panel’s length in a back-to-back configuration (i.e., every measurement point on the top surface of the panel has a corresponding one on the bottom surface of the panel. The five optimal sensing lines have been searched between the 25 lines identified by the centroids of the elements of the inverse mesh (Figure 7). On each of the lines lying on the panel, 38 centroidal locations have been considered as measurement points (whereas 34 centroidal locations have been considered for the lines lying on the stiffeners). The centroids of the inverse elements too close to the supported boundaries, where the presence of the iron bars does not allow the application of the fibre, have not been considered.

**Figure 8:**
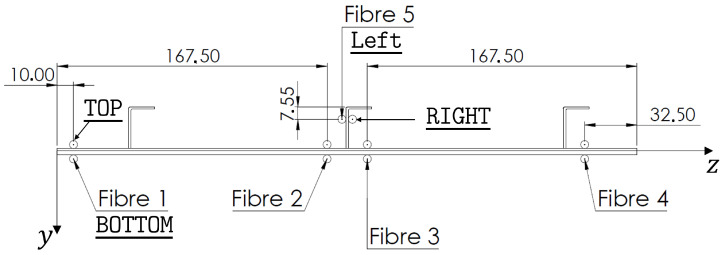
Configuration of the fibre with all dimensions in mm.

In Figure 7 end Figure 8, the selected optimised five lines and the corresponding measurement points are shown. This optimal configuration has been obtained by numerically applying the three methods on the reconstruction of the vertical displacements, along y, of the structure (v). In this phase, the strain inputs and the reference displacements have been computed from the refined FE model of the experiment. All the possible **53,130** configurations of 5 lines out of the 25 possible ones, for each method, have been computed and the relative percent Root Mean Squared Errors ($\%{\mathrm{ERMS}}_{v}$), with respect to the reference vertical displacements of all the 978 nodes of the iFEM mesh, have been collected:

(17) $$\%{\mathrm{ERMS}}_{v}=100\times \sqrt{\frac{1}{978}\sum_{i=1}^{978}{\left(\frac{v_{i}-v_{i}^{\mathrm{ref}}}{v_{\max}^{\mathrm{ref}}}\right)}^{2}}$$

where, $v_{i}$ are the reconstructed displacements, $v_{i}^{\mathrm{ref}}$ are the reference displacements, computed with the refined FE model, and $v_{\max}^{\mathrm{ref}}$ is the maximum value of the reference displacements. The best configuration for each method, that showed the minimum value of the $\%{\mathrm{ERMS}}_{v}$, has been tested on the other methods and the best trade-off configuration has been selected (Figure 7 end Figure 8). For the selected configuration, the numerical $\%{\mathrm{ERMS}}_{v}$ are 3.5, 3.7 and $5.9\times 10^{-6}$ for the iFEM, the MM and the 2-step method respectively. For the MM, two configurations, that resulted in the same $\%{\mathrm{ERMS}}_{v}$, but considering two different sets of retained modes, have been considered to study the influence of the modes’ selection on the local accuracy of the method. The first configuration considers the first 22 modes, that, according to the selection method presented in [15,20], account for the 97.6% of the total strain energy of the static deformation. The second configuration only considers the (1,2,3,8,12) modes, that account for a slightly lower value of the strain energy (96.7%).

## 4. Experimental Results

**Figure 9:**
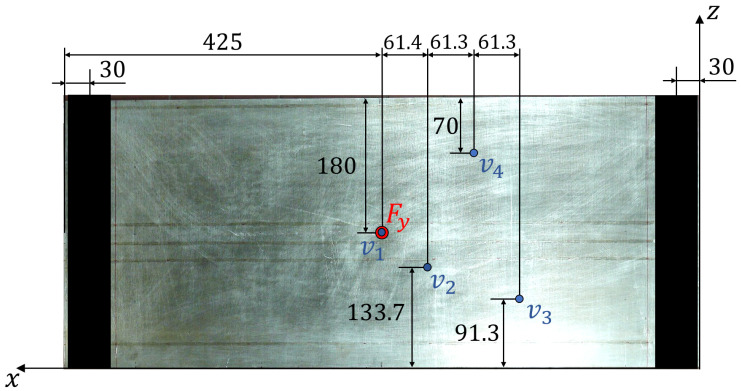
LVDTs’ configuration—The location of the four LVDTs ($v_{1-4}$) on the surface of the panel are shown. All dimensions are expressed in [mm].

The test has been performed by loading the plate as described in Section 3.1. Four LVDTs have been installed on the unstiffened surface of the panel. These sensors measure the transverse displacements along y at the location where the concentrated force is applied ($v_{1}$) and at other three randomly distributed locations on one of the symmetric halves of the panel ($v_{2-4}$). The displacement sensors’ configuration is illustrated in Figure 9. These measured displacements are used as references to evaluate the accuracy of the shape sensing methods.

**Figure 10:**
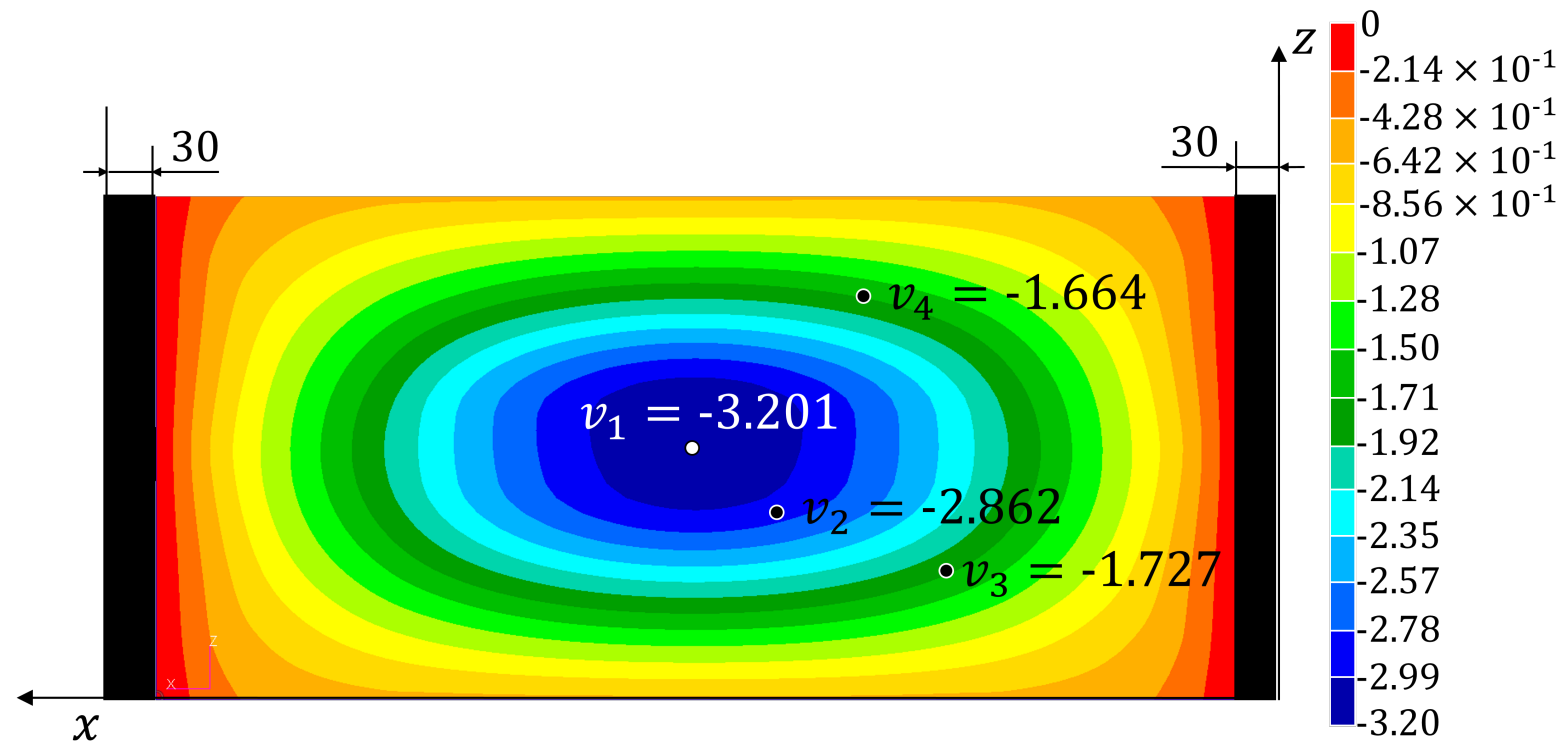
Test 3—Transverse displacement contour for the 2-step method with all dimensions in mm.

**Figure 11:**
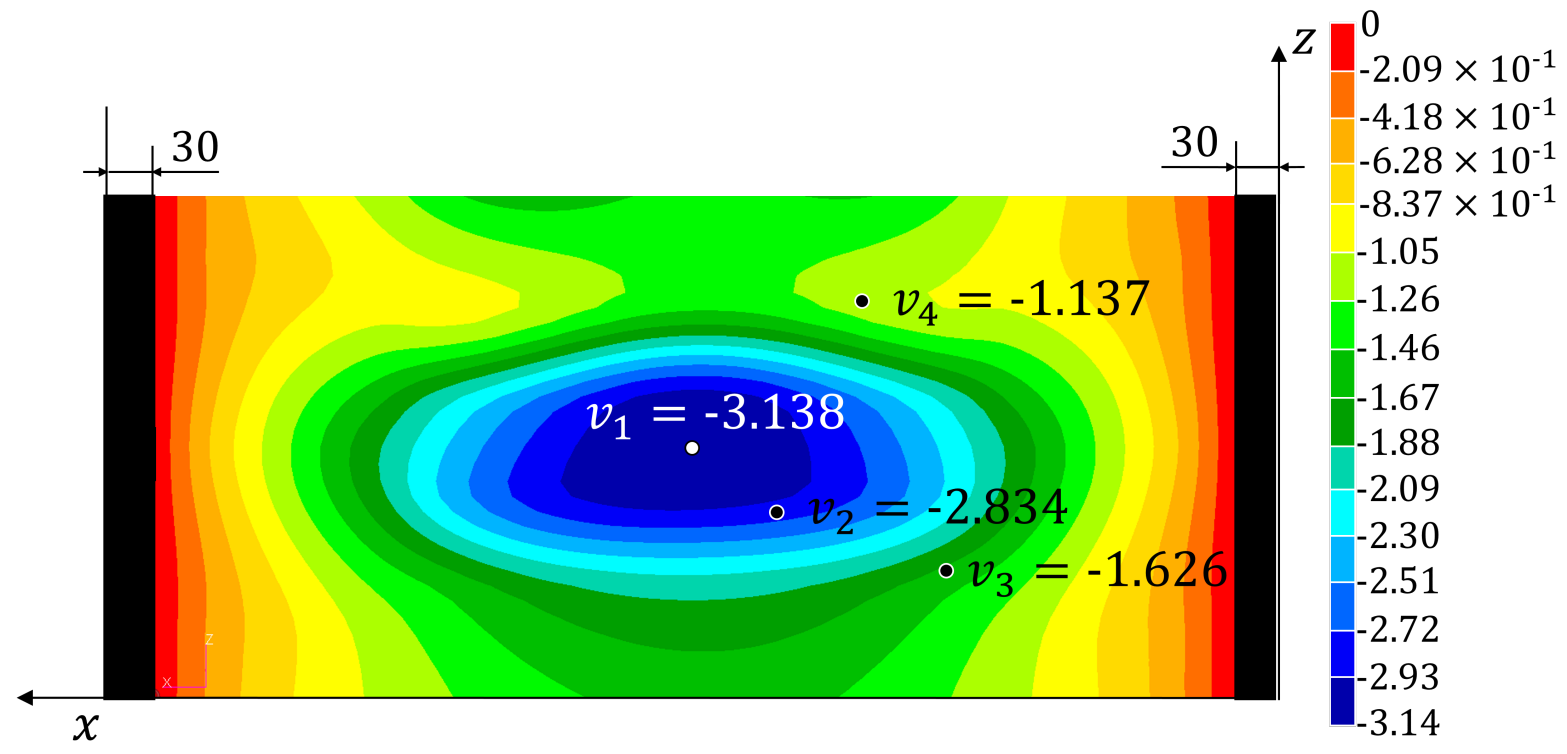
Test 3—Transverse displacement contour for the Model Method (1-22) with all dimensions in mm.

**Figure 12:**
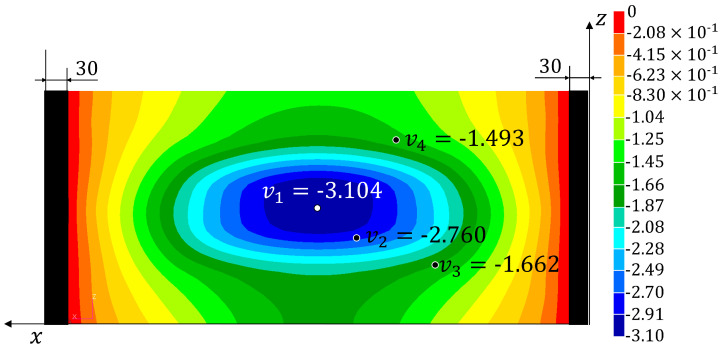
Test 3—Transverse displacement contour for the Modal Method (1,2,3,8,12) with all dimensions in mm.

**Figure 13:**
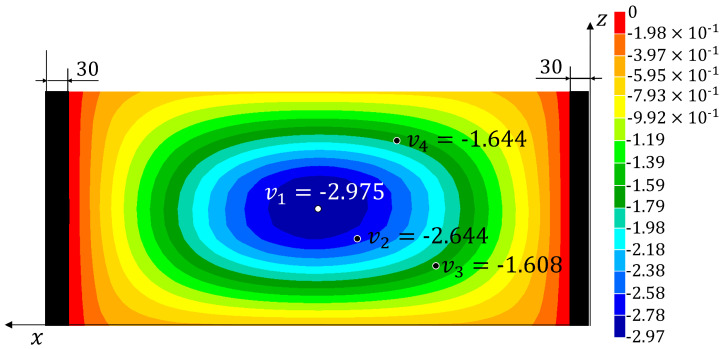
Test 3—Transverse displacement contour for the iFEM with all dimensions in mm.

Three tests have been performed on the panel and the signals from the fibre optic strain sensor and from the LVDTs have been recorded. The strains measured on the fibre are reported in Appendix A. The experimental displacements and loads and those reconstructed using the four monitoring methods are listed in Table 2 (together with the percent errors with respect to the experimental values). The results from the simulation of the test using the refined FE model are also reported (**HF-FEM**). The results show a good reproducibility of the experiment over the three tests and a consequent low level of variability for the experimentally measured quantities and the reconstructed ones. Therefore, the three tests have been considered representative of the experimental behaviour of the structure and no additional tests have been performed. The absolute value of the percent errors, averaged over the three tests ($\%{\bar{\mathrm{Err}}}_{F_{y}}$$\%{\bar{\mathrm{Err}}}_{v_{1-4}}$), have been reported in Table 3. The average error over the four reconstructed displacements ($\%{\bar{\mathrm{Err}}}_{v}$) has also been computed for each method. In Figure 10, Figure 11, Figure 12 and Figure 13, the contours plots for the full transverse displacement field, reconstructed by the four shape sensing methods, are shown for only one representative test, the Test 3.

The average percent errors with respect to the four experimentally measured displacements highlight the impressive accuracy of the **iFEM**. This method shows an average error ($\%{\bar{\mathrm{Err}}}_{v}$) that is 1.5% and a maximum error of 2.2%, thus proving to be highly accurate in the reconstruction of the whole transverse displacement field. Moreover, it is important to remind that this method is able to reach this level of accuracy without the need of any knowledge of the material properties of the structure.

On the other hand, the results prove the sensitivity of the MM to the choice of the retained modes. In fact, the two selected configurations show different distributions of the errors. In particular, the one considering the first 22 modes (**MM** (1–22)), shows a good reconstruction of the first three displacements ($\%{\bar{\mathrm{Err}}}_{v_{1-3}}\leq 7.5\%$) but a really poor reconstruction of the fourth displacement ($\%{\bar{\mathrm{Err}}}_{v_{4}}=31.2\%$). The second configuration (**MM** (1,2,3,8,12)), with only five modes selected, shows a better and more consistent overall accuracy, with an average error of 4.3%. Also in this case, the fourth displacement is reconstructed with lower accuracy ($\%{\bar{\mathrm{Err}}}_{v_{4}}=7.9\%$). These local phenomena, especially for the **MM** (1–22) configuration, can be explained by analysing the working principles of the method. The method tries to reconstruct the deformed shape of the structure as a combination of the modal shapes, by using the strain information as the weights of the combination. Therefore, some mode shapes that exhibit buckles in some areas, if not sufficiently smoothed by the strain information given by the sensors, can bias the overall results obtained by the method, thus generating local inaccuracies. By comparing Figure 11 and Figure 12 with the most accurate reconstruction in Figure 13, it is possible to observe how the transverse displacement field is biased in the areas on the sides of the centre point, where the $v_{4}$ sensor is located.

Finally, the **2-step** approach is able to accurately identify the applied load, with an average error of 2% in the first step of the procedure. The application of the identified load to the refined FE model adds this error to the ones already present in the computation of the displacements using this model. In fact, for each displacement, the percent error is the sum of the percent errors coming from the refined model (**HF-FEM**) and the percent error in the identification of the load. The analysis of these errors shows an overall accuracy that is slightly lower than the one obtained by the **MM**(1,2,3,8,12). Nevertheless, it is important to notice that the accuracy of the 2-step method can be increased by adopting a refined model that is more representative of the experimental set-up, thus reducing the error coming from the model’s inaccuracy. Moreover, the 2-step method is the only one, within this study, that can simultaneously reconstruct the displacements and the applied load.

## 5. Conclusions

This paper presents a comparative study on the shape sensing of a stiffened aluminium panel. Three shape sensing methods are considered, the iFEM, the Modal Method (MM) and the 2-step method, the latter being able to simultaneously identify the applied load on the structure. The panel, tested in a laboratory environment with a simply supported configuration, is instrumented with load, displacement and optical fibre strain sensors to collect the data necessary for the application hlof the three methods and for the assessment of the relative accuracy.

The results of the analysis show the superior accuracy of the iFEM in the reconstruction of the vertical displacements experienced by the panel. The MM, on the other hand, leads to an averagely good reconstruction of the displacement field, but it shows some local inaccuracies. This difficulty is influenced by the selection of the modes for the application of the method. In fact, in some areas, the method is biased by the used mode shapes. Finally, the 2-step method is able to effectively identify the load and to achieve a good level of global shape sensing accuracy. The experimental test also demonstrates that the accuracy of the 2-step’s shape sensing is strongly related to the ability of the refined model, used to reconstruct the shape, to actually reproduce the behaviour of the monitored structure. The results of the comparative investigation provides important guidelines for the application of shape sensing methodologies to stiffened structures with fibre optic strain sensors. iFEM is highly accurate and its performances are even more relevant considering that the method does not need the knowledge of the material properties. Both the Modal Method and the 2-step method are slightly less accurate and require the knowledge of the material properties. Moreover, MM can be negatively affected by the selected mode shapes. On the other hand, the 2-step method proves to be an important and complete tool for the structural monitoring, being able to simultaneously address the load identification and displacement reconstruction problems. Future work should explore the capabilities of this method on more complex load configurations. Moreover, the robustness of the sensors’ configuration with respect to the different deformed shapes, that the monitored structure could assume due to diverse loading conditions, should be assessed for all the three methods.

## Figures and Tables

**Table 1:** Mechanical properties of the AL-Li alloy.

	Al-Li Alloy
E[MPa]	75,958
ν	0.300
$\rho \;[g/{\mathrm{cm}}^{3}]$	2.78

**Table 2:** Shape sensing and load identification results for the stiffened panel. In parenthesis, the percentage errors with respect to the experimental values are reported. The errors are computed considering the absolute value of the displacements.

	Experimental	HF-FEM	2-Step	MM (1–22)	MM (1,2,3,8,12)	iFEM
	**Test 1**					
$F_{y}\;\left[N\right]$	−865.0		−883.1			
($\%{\mathrm{Err}}_{F_{y}}$)			(+2.1%)			
$v_{1}\;\left[\mathrm{mm}\right]$	−3.000	−3.078	−3.142	−3.081	−3.047	−2.916
($\%{\mathrm{Err}}_{v_{1}}$)		(+2.6%)	(+4.7%)	(+2.7%)	(+1.6%)	(−2.8%)
$v_{2}\;\left[\mathrm{mm}\right]$	−2.644	−2.752	−2.809	−2.823	−2.705	−2.624
($\%{\mathrm{Err}}_{v_{2}}$)		(+4.1%)	(+6.2%)	(+6.8%)	(+2.3%)	(−0.8%)
$v_{3}\;\left[\mathrm{mm}\right]$	−1.614	−1.660	−1.695	−1.653	−1.627	−1.641
($\%{\mathrm{Err}}_{v_{3}}$)		(+2.9%)	(+5.0%)	(+2.4%)	(+0.8%)	(+1.7%)
$v_{4}\;\left[\mathrm{mm}\right]$	−1.610	−1.600	−1.633	−1.058	−1.475	−1.562
($\%{\mathrm{Err}}_{v_{4}}$)		(−0.6%)	(+1.4%)	(−34.3%)	(−8.4%)	(−3.0%)
	**Test 2**					
$F_{y}\;\left[N\right]$	−882.0		−899.9			
($\%{\mathrm{Err}}_{F_{y}}$)			(+2.0%)			
$v_{1}\;\left[\mathrm{mm}\right]$	−3.002	−3.138	−3.202	−3.139	−3.104	−2.973
($\%{\mathrm{Err}}_{v_{1}}$)		(+4.5%)	(+6.7%)	(+4.6%)	(+3.4%)	(−1.0%)
$v_{2}\;\left[\mathrm{mm}\right]$	−2.634	−2.806	−2.863	−2.825	−2.761	−2.657
($\%{\mathrm{Err}}_{v_{2}}$)		(+6.5%)	(+8.7%)	(+7.3%)	(+4.8%)	(+0.9%)
$v_{3}\;\left[\mathrm{mm}\right]$	−1.603	−1.693	−1.727	−1.605	−1.662	−1.631
($\%{\mathrm{Err}}_{v_{3}}$)		(+5.6%)	(+7.7%)	(+0.1%)	(+3.7%)	(+1.7%)
$v_{4}\;\left[\mathrm{mm}\right]$	−1.613	−1.631	−1.644	−1.130	−1.481	−1.638
($\%{\mathrm{Err}}_{v_{4}}$)		(+1.1%)	(+1.9%)	(−29.9%)	(−8.2%)	(+1.5%)
	**Test 3**					
$F_{y}\;\left[N\right]$	−882.0		−899.7			
($\%{\mathrm{Err}}_{F_{y}}$)			(+2.0%)			
$v_{1}\;\left[\mathrm{mm}\right]$	−3.004	−3.138	−3.201	−3.138	−3.104	−2.975
($\%{\mathrm{Err}}_{v_{1}}$)		(+4.5%)	(+6.6%)	(+4.5%)	(+3.3%)	(−1.0%)
$v_{2}\;\left[\mathrm{mm}\right]$	−2.649	−2.806	−2.862	−2.834	−2.760	−2.644
($\%{\mathrm{Err}}_{v_{2}}$)		(+5.9%)	(+8.0%)	(+7.0%)	(+4.2%)	(−0.2%)
$v_{3}\;\left[\mathrm{mm}\right]$	−1.622	−1.693	−1.727	−1.626	−1.662	−1.608
($\%{\mathrm{Err}}_{v_{3}}$)		(+4.4%)	(+6.5%)	(+0.2%)	(+2.5%)	(−0.9%)
$v_{4}\;\left[\mathrm{mm}\right]$	−1.609	−1.631	−1.664	−1.137	−1.493	−1.644
($\%{\mathrm{Err}}_{v_{4}}$)		(+1.4%)	(+3.4%)	(−29.3%)	(−7.2%)	(+2.2%)

**Table 3:** Absolute value of the percent errors of the reconstructed quantities averaged over the three tests.

	2-Step	MM (1–22)	MM (1,2,3,8,12)	iFEM
$\%{\bar{\mathrm{Err}}}_{F_{y}}$	2.0%			
$\%{\bar{\mathrm{Err}}}_{v_{1}}$	6.0%	3.9%	2.8%	1.6%
$\%{\bar{\mathrm{Err}}}_{v_{2}}$	7.7%	7.0%	3.8%	0.6%
$\%{\bar{\mathrm{Err}}}_{v_{3}}$	6.4%	0.9%	2.3%	1.4%
$\%{\bar{\mathrm{Err}}}_{v_{4}}$	2.3%	31.2%	7.9%	2.2%
$\%{\bar{\mathrm{Err}}}_{v}$	5.6%	10.8%	4.2%	1.5%

**Figure A1:**
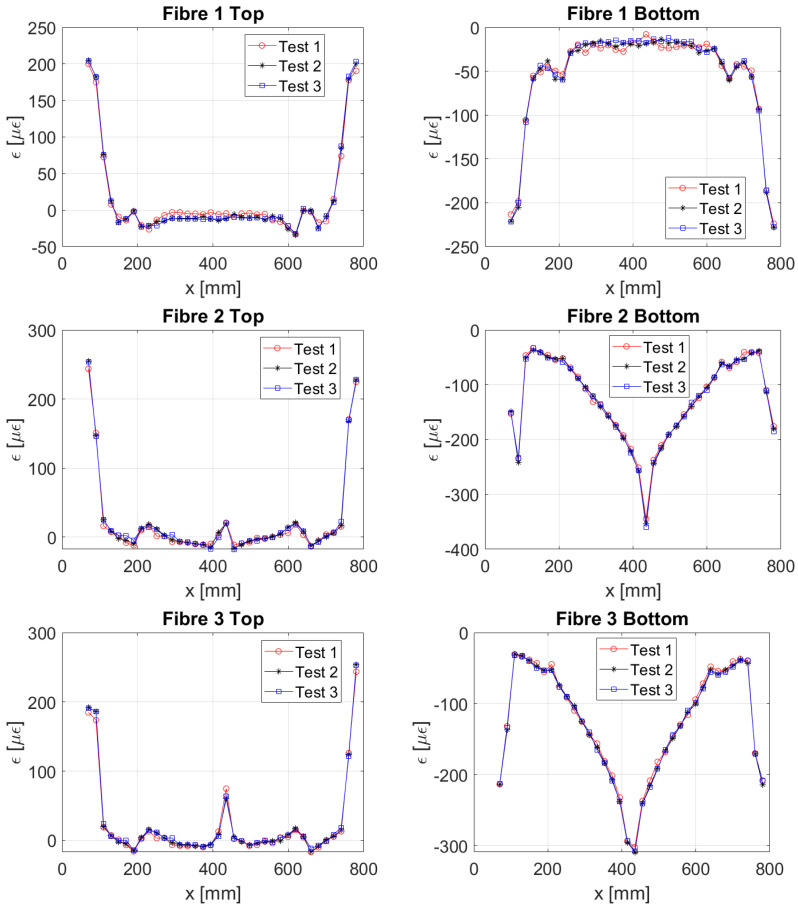
Experimental strains from the stiffened panel—The numbering of the fibres is the one reported in Figure 8.

**Figure A2:**
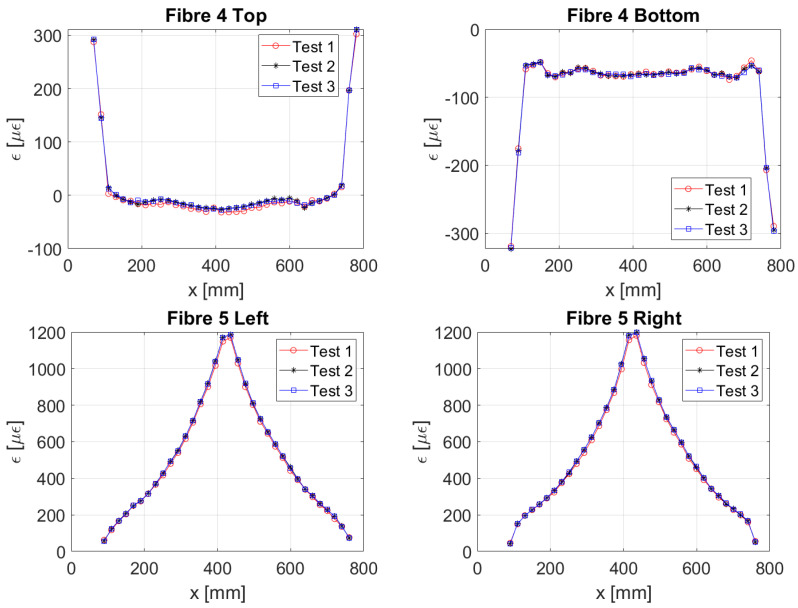
Experimental strains from the stiffened panel—The numbering of the fibres is the one reported in Figure 8.

## Data Availability

Not applicable.
